# *Notes from the Field*: Seroprevalence of Highly Pathogenic Avian Influenza A(H5) Virus Infections Among Bovine Veterinary Practitioners — United States, September 2024

**DOI:** 10.15585/mmwr.mm7404a2

**Published:** 2025-02-13

**Authors:** Jerome Leonard, Elizabeth J. Harker, Christine M. Szablewski, Sara F. Margrey, K. Fred Gingrich, Keyana Crossley, Emily Fletcher, Claire J. McCreavy, Sabrina Weis-Torres, Dennis Wang, Emma K. Noble, Min Z. Levine, H. Pamela Pagano, Crystal Holiday, Feng Liu, Stacie Jefferson, Zhu-Nan Li, F. Liaini Gross, Carrie Reed, Sascha Ellington, Alexandra M. Mellis, Samantha M. Olson

**Affiliations:** ^1^Influenza Division, National Center for Immunization and Respiratory Diseases, CDC; ^2^Epidemic Intelligence Service, CDC; ^3^Ohio Department of Health; ^4^American Association of Bovine Practitioners, Ashland, Ohio; ^5^Office of Safety, Security, and Asset Management, CDC; ^6^Division of Viral Hepatitis, National Center for HIV, Viral Hepatitis, STD, and Tuberculosis Prevention, CDC; ^7^Coronavirus and Other Respiratory Viruses Division, National Center for Immunization and Respiratory Diseases, CDC.

**Summary**

**What is already known about this topic?**

Highly pathogenic avian influenza (HPAI) A(H5) virus infections have been detected in humans exposed to infected dairy cattle.

**What is added by this report?**

Public health officials conducted a serosurvey among 150 bovine veterinary practitioners. Three practitioners had evidence of recent infection with HPAI A(H5) virus, including two without exposures to animals with known or suspected HPAI A(H5) virus infections and one who did not practice in a U.S. state with known HPAI A(H5) virus–infected cattle.

**What are the implications for public health practice?**

These findings suggest the possible benefit of systematic surveillance for rapid identification of HPAI A(H5) virus in dairy cattle, milk, and humans who are exposed to cattle to ensure appropriate hazard assessments.

The current outbreak of highly pathogenic avian influenza (HPAI) A(H5) clade 2.3.4.4.b viruses, genotype B3.13, among dairy cattle was first detected in March 2024 (*1*), with human cases of HPAI A(H5) among dairy farm workers identified beginning in April (*2*). Farm workers and bovine veterinary practitioners working with HPAI A(H5) virus–infected cattle are at increased risk for HPAI A(H5) exposure; in the current outbreak, most human infections with HPAI A(H5) have been mild and were detected through enhanced surveillance of persons working with affected animals (*2*).

## Investigation and Outcomes

To investigate the prevalence of recent HPAI A(H5) infection among U.S. bovine veterinary practitioners, CDC conducted an HPAI A(H5) serosurvey (September 12–13, 2024) with a target enrollment of 150 bovine veterinary practitioners with cattle exposure in the previous 3 months and assessed their exposures since January 2024. Practitioners were recruited in-person at an annual veterinary conference and through emails to conference attendees. Participation was anonymous, and participants received a $50 USD gift card as compensation after the blood draw. At the time of this serosurvey, HPAI A(H5) in dairy cattle had been detected in 14 U.S. states, with four human cases in persons with dairy cattle exposure in three states.* This serosurvey was reviewed and approved by the Ohio Department of Health institutional review board and was conducted consistent with applicable federal law and CDC policy.^†^

All 150 surveyed practitioners were serologically tested for antibodies to recent HPAI A(H5) virus infection (*3*). Participants reported their primary practice in 46 U.S. states (143) and Canada (seven). Among all survey participants, 82 (55%) practiced in states with HPAI A(H5) virus–positive dairy herds, and 25 (17%) worked with dairy cattle with known or suspected HPAI A(H5) infection.^§^

Three (2%; 95% CI = 0.7%–5.7%) survey participants had antibodies to HPAI A(H5)^¶^ suggestive of recent HPAI A(H5) infection; all were U.S.-based practitioners. None of the practitioners with positive serology results reported respiratory or influenza-like symptoms, including conjunctivitis** nor had any received testing for influenza since January 2024.

All three practitioners with positive serology results provided care to multiple animals,^††^ including dairy cattle; two also provided care to nondairy cattle, one provided care to poultry, and one worked at livestock markets. None worked with dairy cattle with known or suspected HPAI A(H5) virus infection; however, one practitioner did work with HPAI A(H5) virus–positive poultry. Two of the participants with a positive serologic test result reported practicing in multiple U.S. states, and two practiced in states with known HPAI A(H5) infection among cattle (Figure). However, one reported providing veterinary care to dairy cattle only in Georgia and to nondairy cattle in South Carolina; these states had not previously reported HPAI A(H5) infection in dairy cattle (*1*). All reported wearing gloves or a clothing cover when providing veterinary care to cattle (including a variety of clinical activities, such as pregnancy checking or surgery)^§§^; none reported wearing respiratory or eye protection.

**FIGURE:**
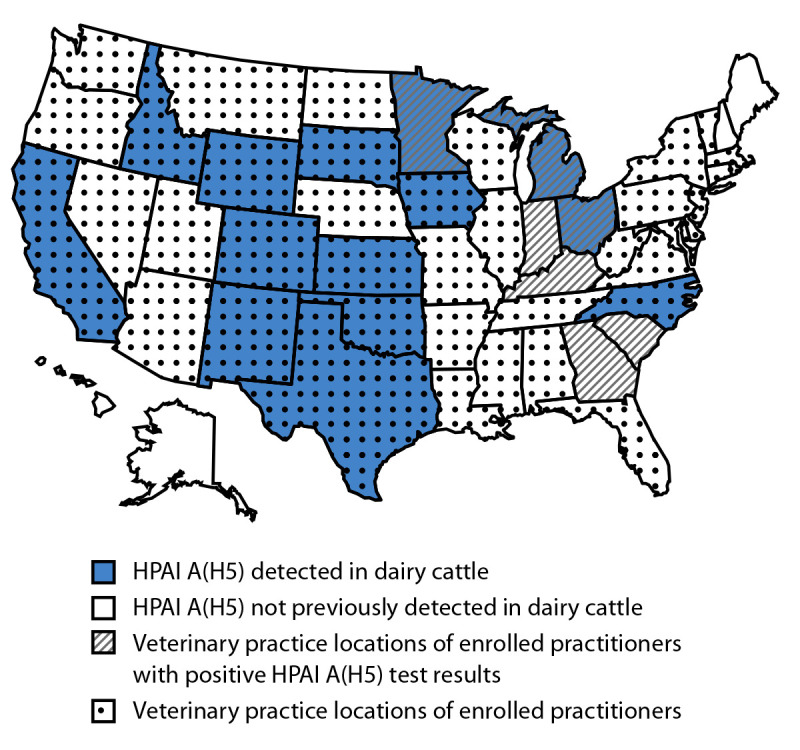
States with serosurvey-enrolled bovine veterinary practitioners* and states reporting highly pathogenic avian influenza A(H5) infections in dairy cattle — United States, September 2024 **Abbreviation:** HPAI = highly pathogenic avian influenza. * Two practitioners with a positive serologic HPAI A(H5) test result indicating recent infection practiced in multiple states.

## Preliminary Conclusions and Actions

Among 150 bovine veterinary practitioners, three had evidence of recent infection with HPAI A(H5) virus, including one who only practiced in two states (Georgia and South Carolina) with no known HPAI A(H5) virus infection in cattle and no reported human cases (*1*,*2*); this practitioner reported no exposures to animals with known or suspected HPAI A(H5) virus infections. These findings suggest that there might be HPAI A(H5) virus–infected dairy cattle in states where infection in dairy cattle has not yet been identified, highlighting the importance of rapid identification of infected dairy cattle through herd and bulk milk testing as recently announced by the U.S. Department of Agriculture.^¶¶^

No practitioners with positive HPAI A(H5) serology results in this study reported influenza-like symptoms, including conjunctivitis. Detection of HPAI A(H5) antibodies in persons without reported symptoms suggests that surveillance of symptomatic exposed workers might underestimate human infection. CDC recently recommended offering the influenza antiviral oseltamivir as postexposure prophylaxis or treatment and HPAI A(H5) molecular testing to asymptomatic workers with high exposure*** to infected animals (*4*). Continued efforts to disseminate these guidelines are important for persons with exposure to dairy cattle, including bovine veterinary practitioners.

No seropositive practitioner knew that they were working with dairy cattle with known or suspected HPAI A(H5) infection. None of the seropositive practitioners reported wearing respiratory or eye protection while providing veterinary care to cattle. Neither respiratory or eye protection is recommended when working with uninfected animals in regions without confirmed cases; however, safety goggles and a respirator are recommended when working with uninfected animals in regions where there are confirmed or potentially infected animals (*4*). HPAI A(H5) virus is known to be present in high concentrations in milk produced by infected cattle, introducing infection risk through respiratory, ocular, and gastrointestinal exposure (*5*). Continued systematic surveillance of livestock and milk could aid in appropriate occupational hazard assessment.^†††^

Since the time that this serosurvey was conducted, the HPAI A(H5) outbreak has expanded to include 67 confirmed human cases, including 40 with dairy cattle exposure (*1*,*2*). These data highlight the possible benefit of national seroprevalence assessments of recent HPAI A(H5) infection among practitioners at increased risk for exposure, which might help assess occupational risk in states without confirmed HPAI A(H5) virus detections in dairy cattle.

## Footnotes

* As of September 13, a total of 14 cases of HPAI A(H5) virus infection in humans had been detected, four of whom had exposure to dairy cattle, nine to poultry, and one to an unidentified source. States with reported dairy herd infections of HPAI A(H5) as of September 2024 include the following 14 states: California, Colorado, Idaho, Iowa, Kansas, Michigan, Minnesota, New Mexico, North Carolina, Ohio, Oklahoma, South Dakota, Texas, and Wyoming. States with reported human HPAI A(H5) virus infections with dairy cattle exposures (as of September 2024) include Colorado, Michigan, and Texas.

† 45 C.F.R. part 46.102(l)(2), 21 C.F.R. part 56; 42 U.S.C. Sect. 241(d); 5 U.S.C. Sect. 552a; 44 U.S.C. Sect. 3501 et seq.

§ Suspected infection for cattle and poultry was defined as having clinical suspicion or signs or symptoms of HPAI A(H5) virus infection without confirmatory testing.

¶ A positive serologic test result is defined as antibody titers ≥40 by the microneutralization assay and titers ≥40 by the hemagglutinin inhibition assay against a wild type influenza A(H5N1) 2.3.4.4b A virus. https://www.who.int/teams/global-influenza-programme/avian-influenza/case-definitions

** Practitioners were asked, “Have you had any respiratory or flu-like symptoms since January 2024?” Those who responded affirmatively were asked about specific symptoms including fever; feverishness or chills; cough; fatigue; sore throat; runny or stuffy nose; sneezing; nausea or vomiting; diarrhea; headache; rash; muscle or body aches; red, draining, or itching eyes (and specifically conjunctivitis); difficulty breathing or shortness of breath; and seizures.

†† Practitioners with evidence of HPAI A(H5) virus infection also reported exposure to goats or sheep (three), swine (two), and cats (three). One practitioner reported providing veterinary care to another (unspecified) animal not listed in the survey (i.e., not dairy cattle; other cattle; swine, pigs, or hogs; alpacas or llamas; poultry; wild birds including wild waterfowl; goats or sheep; or cats).

§§ Practitioners reported the following activities when working with dairy cattle: intervening during calving or maternity care (two); surgery (two); mammary exam or treatment administration (one); feeding (two); drenching, tubing, or administering oral medications (one); administering vaccines or injectable medications including intravenous fluids (two); podiatry (one); pregnancy checking (three); and postmortem examinations or necropsies (two). Use of personal protective equipment varied based on the clinical activity.

¶¶
https://www.aphis.usda.gov/news/agency-announcements/usda-builds-actions-protect-livestock-public-health-h5n1-avian-influenza

*** Defined as contact with living or dead animals with confirmed or potential HPAI A(H5) infection (e.g., poultry culling operations or work in sick pens) or contact with raw milk, other secretions, udders, or viscera from a farm with confirmed or potentially infected animals (e.g., work in a milking parlor, raw milk processing, and some slaughterhouse work).

†††
https://www.aphis.usda.gov/sites/default/files/20241205-federal-order-final.pdf
